# Multiple potential targets of opioids in the treatment of acute respiratory distress syndrome from COVID‐19

**DOI:** 10.1111/jcmm.15927

**Published:** 2020-11-19

**Authors:** Cosmin Andrei Cismaru, Gabriel Laurentiu Cismaru, Seyed Fazel Nabavi, Mostafa Ghanei, Claudia Cristina Burz, Seyed Mohammad Nabavi, Ioana Berindan Neagoe

**Affiliations:** ^1^ Research Center for Functional Genomics, Biomedicine and Translational Medicine The “Iuliu Hatieganu” University of Medicine and Pharmacy Cluj‐Napoca Romania; ^2^ Department of Functional Sciences, Immunology and Allergology The “Iuliu Hatieganu” University of Medicine and Pharmacy Cluj‐Napoca Romania; ^3^ Department of Internal Medicine Cardiology‐Rehabilitation The “Iuliu Hatieganu” University of Medicine and Pharmacy Cluj‐Napoca Romania; ^4^ Applied Biotechnology Research Center Baqiyatallah University of Medical Sciences Tehran Iran; ^5^ Chemical Injuries Research Center Systems Biology and Poisoning Institute Baqiyatallah University of Medical Sciences Tehran Iran; ^6^ The Functional Genomics Department The Oncology Institute “Prof. Dr. Ion Chiricuta” Cluj‐Napoca Romania

**Keywords:** ARDS, COVID‐19, cytokine storm, dyspnoea, immunomodulation, opioids

## Abstract

COVID‐19 can present with a variety of clinical features, ranging from asymptomatic or mild respiratory symptoms to fulminant acute respiratory distress syndrome (ARDS) depending on the host's immune responses and the extent of the associated pathologies. This implies that several measures need to be taken to limit severely impairing symptoms caused by viral‐induced pathology in vital organs. Opioids are most exploited for their analgesic effects but their usage in the palliation of dyspnoea, immunomodulation and lysosomotropism may represent potential usages of opioids in COVID‐19. Here, we describe the mechanisms involved in each of these potential usages, highlighting the benefits of using opioids in the treatment of ARDS from SARS‐CoV‐2 infection.

## 1 INTRODUCTION

COVID‐19 currently represents an ongoing global threat as the development of ARDS during the course of disease may be observed in more than 40% of patients with pneumonia from SARS‐CoV‐2 infection.^1^ ARDS from COVID‐19 requires early recognition and a comprehensive management as it can have worse outcomes than ARDS from other causes. Pulmonary fibrosis, thrombotic microangiopathy, pleuritic pain and worsening of respiratory symptoms appear to be factors of a more severe course of disease from the increased inflammatory responses induced by SARS‐CoV‐2 infection.^2^ While opioids can be used for the subjective perception of ARDS from COVID‐19,^3^ several other properties of opioids may address the physiopathological mechanisms involved in ARDS development and limit essential steps of the viral infectious cycle. Morphine is an organic heteropentacyclic tertiary amino compound being a morphinane alkaloid with potent analgesic and psychoactive properties. It is most abundant in opium poppy (*Papaver somniferum* L)^4^ and acts directly on the central nervous system (CNS) to alleviate pain. By its chemical structure, morphine derives from a hydride of a morphinan, being a conjugate base of a morphine (1+). Specific opiate receptors for morphine are found in brain and control different functions, including analgesia, euphoria, sedation, anxiolysis and respiratory depression.^5^ The opioid receptors have been previously described and are represented by miu(µ), delta(δ) and kappa(κ) receptors. While many of them are involved in the perception of pain and dyspnoea, opioid receptors are also found in the digestive system inhibiting bowel movement and on the surface of immune cells having immunomodulatory effects.^6^ Opioids are widely used for treating moderate to severe pain in patients who require potent analgesia, mostly in the setting of acute trauma, childbirth, invasive procedures and chronic end‐stage illnesses.^7^ While strong opioids are potent analgesics, less strong representatives such as codeine and DHC have more pronounced effects on cough, ^8^ diarrhoea ^9^ and dyspnoea.^10^

## 2 OPIOIDS AND DYSPNOEA

Opiate drugs have long been known to exert effects on the respiratory system. These include reducing the respiratory response to CO_2_,^11^ hypoxia,^12^ inspiratory flow‐resistive loading^13^ and exercise,^14^ with overdoses being capable of producing respiratory depression.^15^ Opioid receptors from various areas of the CNS and the cardio‐respiratory systems seem responsible for the mediation of the mechanisms of their antidyspnoeic effects.^16^, ^17^, ^18^ Moreover, in the palliation of dyspnoea, opioids are the only agents with sufficient pharmacological evidence.^10^, ^19^, ^20^ These are some of the arguments which led to the usage of opioids in the palliation of dyspnoea, being recommended by European and US therapeutic guidelines.^21^, ^22^ Treatment of dyspnoea outside oncological pathology such as the chronic obstructive pulmonary diseases (COPD)^23^, ^24^, ^25^ is emerging as an indication in professional society guidelines such as the GOLD.^26^ While special precautions regarding morphine usage in patients with severe renal insufficiency, the dosage and dosage intervals require adaptation to the renal function for all µ‐opioids due to their renal elimination.^27^, ^28^, ^29^ In patients without severely impaired kidney function, they can be used in both opioid‐naïve and opioid‐treated patients without relevant breath depression, impaired oxygenation or increase in CO2 concentration.^30^ While dyspnoea and anxiety occur most often as associated events, opioids have been known to reduce the subjective perception of breathlessness, mainly by increasing the amplitude of breaths and limiting hyperventilation and tachypnoea while exerting anxiolytic effects. No opioid is superior to another in treating dyspnoea and oxycodone, and hydromorphone and fentanyl can be used for this purpose, while dihydrocodeine, diamorphine, oral and paranteral morphine have the most evidence for this use.^23^, ^31^, ^32^, ^33^

Opioid‐naïve patients require smaller doses of opioids for dyspnoea than for the palliation of pain. However, patients already on opioid treatment will require increases of the baseline dosage of up to 25%‐50% for the palliation of dyspnoea.^34^ This is associated with the advantages of significant decrease in the intensity of dyspnoea, tachypnoea and no higher risk of respiratory depression but with the drawback of unwanted side‐effects such as initial nausea and constipation.^35^

## 3 OPIOIDS AND IMMUNOMODULATION

**FIGURE 1:**
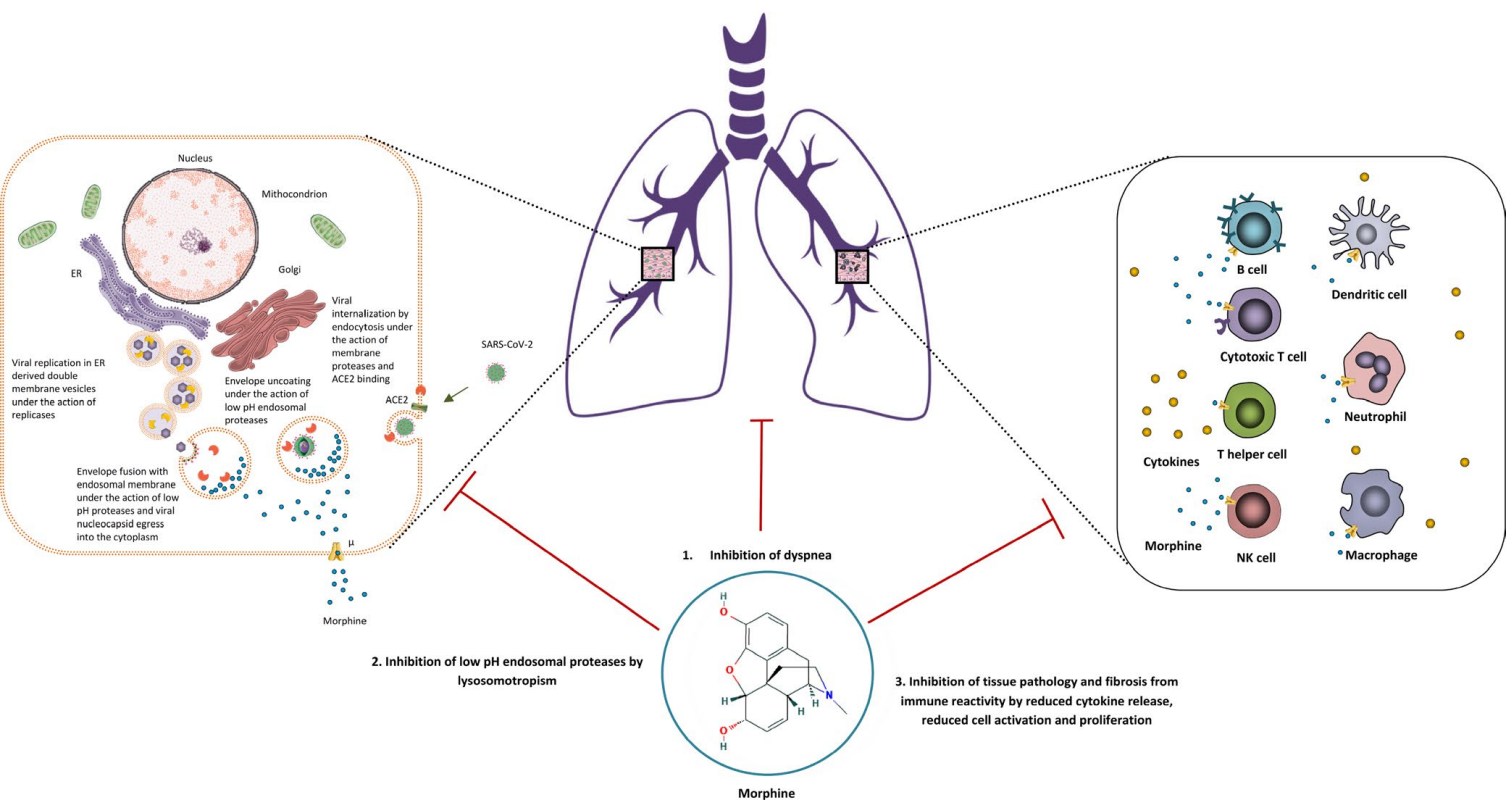
Schematic representation of multiple potential targets the opioid morphine in COVID‐19

Opioid receptors are present on various cells of the immune system (eg lymphocytes, monocytes, macrophages and neutrophils) exerting inhibitory effects on lymphocyte proliferation and cytokine release.^6^, ^36^ (Figure 1). In vitro and in vivo animal experiments have shown a wide spectrum of effects of morphine such as anti‐inflammatory, antifibrotic, antitumour, cardioprotective and renoprotective.^37^, ^38^, ^39^ These effects could counteract the excessive inflammation, fibrosis and cardiac and renal pathology associated with COVID‐19 as much of its pathology is associated with the dysregulation of the renin‐angiotensin system (RAS) from antagonization of angiotensin‐converting enzyme 2 (ACE2).^40^ Besides, activation of the inflammasome with subsequent adaptative immune responses and cytokine release syndrome or the cytokine storm is seen in SARS‐CoV^41^ as well as in SARS‐CoV‐2 pathogenesis.^42^ While a reduced pathogenesis from cytokine release and inflammatory cell infiltration in the lungs has been observed with the immunomodulatory effect of opioids in various viral infections, immunosuppression from extended use is not to be overlooked.^43^, ^44^ In COVID‐19, the distribution of ACE2 receptors mostly in lung tissue but also in heart, kidney, brain and endothelia makes the respiratory tract carry the most important load of viral‐induced pathology and the other organs expressing ACE2 the second most affected by the viral replication and the hosts’ response against it. Most of the pathology is induced by the immune response against the invading pathogen rather than the infection itself as the virus uses the replicative machinery of the cell followed by budding for its egress rather than cell lysis.^45^ The host cell death can occur if excess Ang II is produced leading to direct apoptosis^46^ and when excess protein is produced with endoplasmic reticulum (ER) stress, unfolded protein response (UPR) and subsequent apoptosis.^47^ Whether a link exists between the antifibrotic and antiinflamatory effects of the depressor arm of RAS with the ACE2 receptors and the antiinflamatory and antifibrotic effects from stimulating opioid receptors in the immune system remains elusive but could represent a future research direction as both receptors are abundant in the brain and peripheral organs and exert seemingly inter‐related effects. This could also support the usage of opioids for addressing neurological manifestations of COVID‐19 which are not rare occurrences.^48^, ^49^, ^50^

## 4 OPIOIDS AND LYSOSOMOTROPISM

Most amine drugs are naturally occurring alkaloids or synthesis compounds containing the amino group. Being weak bases, they have the ability of lysosomotropism.^51^ This translates in the property of accumulating in acidic lysosomes in concentrations several hundred times higher than in the cytoplasm increasing lysosomal pH by becoming trapped following protonation in the ion‐trapping process.^51^, ^52^ The discoverer of lysosomes, Christian de Duve, who was awarded the Nobel Prize for Physiology and Medicine in 1974 together with George Emil Palade and Albert Claude for their work on the functional organization of the cell, elegantly described in a commentary the theoretical basis for lysosomotropism and physiopathological and clinical implications of this process.^53^ In most infections with enveloped viruses, the viral pathogens depend on the low‐pH endosomal hydrolases to uncoat their envelope and fuse with the lysosomal membrane, but such processes may be disrupted by aminic drugs that increase the lysosomal pH, exposing the virus to the organelle's degradative enzymes.^54^ Since morphine, an aminic drug and the alkaloid of morphinane have long been shown to accumulate in lysosomes having lysosomotropic properties,^55^ we may assert that it could have an inhibitory effect on lysosomal acidification along other opioid alkaloids. This is supported by the observations on the lysosomotropic effects of morphine on another enveloped RNA virus the human immunodeficiency virus—HIV‐1^56^ and of multiple other lysosomotropic drugs on the hepatitis C virus—HCV, an enveloped RNA virus.^57^ Moreover, loperamide, an opioid receptor agonist and weak base, was shown to become trapped and increase lysosomal pH being lysosomotropic^58^ and inhibiting MERS‐CoV and SARS‐CoV‐2 replication in cell culture.^59^, ^60^, ^61^ The lysosomotropic effect of opiate drugs could therefore be exploited in COVID‐19 for the inhibition of viral uncoating in the endosomal internalization pathway as this is a common entry route for coronaviruses^62^

## 5 CONCLUSIONS

Triple effects of opioids could potentially be exploited in COVID‐19, such as treating dyspnoea, inhibiting the cytokine storm and disrupting lysosomal acidification with effects on both the viral infectious cycle and on the host's response to the infection. Due care should be taken when using opioid drugs as they have a high potential for addiction, with physical and psychological tolerance and dependence often occurring. However, in emergency situations such as ARDS from COVID‐19 at the currently indicated dosages for treating dyspnoea, the benefits of using opioids could overcome the unpleasant side‐effects, addressing both the viral infectious cycle and the host's response to the viral‐induced pathogenesis. These represent arguments to support the perspective of using opioids in addressing the clinical manifestations and pathogenesis associated with the ARDS from the SARS‐CoV‐2 infection.

## CONFLICT OF INTEREST

The authors declare no competing interests.

## AUTHOR CONTRIBUTIONS

**Andrei Cismaru:** Conceptualization (equal); Writing‐original draft (equal). **Gabriel Cismaru:** Conceptualization (equal); Writing‐review & editing (equal). **Seyed Fazel Nabavi:** Conceptualization (equal); Writing‐review & editing (equal). **Seyed Mohammad Nabavi:** Conceptualization (equal); Writing‐review & editing (equal). **Mostafa Ghanei:** Writing‐review & editing (equal). **Claudia Cristina Burz:** Writing‐review & editing (equal). **Ioana Berindan‐Neagoe:** Conceptualization (equal); Supervision (equal); Writing‐review & editing (equal).

## Data Availability

Data sharing is not applicable to this article as no new data were created or analyzed in this study.
